# IDO1 Modulates the Sensitivity of Epithelial Ovarian Cancer Cells to Cisplatin through ROS/p53-Dependent Apoptosis

**DOI:** 10.3390/ijms231912002

**Published:** 2022-10-09

**Authors:** Houmei Wang, Yuanyuan Luo, Rui Ran, Xinya Li, Hongjian Ling, Fang Wen, Tinghe Yu

**Affiliations:** Laboratory of Obstetrics and Gynecology, The Second Affiliated Hospital, Chongqing Medical University, Chongqing 400010, China

**Keywords:** cisplatin, indoleamine 2,3-dioxygenase 1, ovarian cancer, p53, reactive oxygen species

## Abstract

Indoleamine 2,3-dioxygenase 1 (IDO1) is a heme-containing dioxygenase that may play a part in chemoresistance in ovarian cancer. However, its role in cisplatin (DDP) resistance is unclear. Here, the expression level of IDO1 in tumors in platinum-resistant (*n* = 22) and -sensitive (*n* = 46) ovarian cancer patients was determined, and then how IDO1 modulated DDP resistance was explored in vitro and in vivo. The IDO1 expression level in platinum-resistant patients was higher than that in -sensitive patients, and a higher IDO1 level was correlated with poor prognosis in type II cancer patients. Up-regulating IDO1 decreased DDP-induced apoptosis in SKOV3 cells via inhibiting the ROS/p53 cell-death pathway, thereby attenuating cytotoxicity of DDP. Silencing IDO1 enhanced p53-dependent apoptosis by increasing ROS accumulation, thereby enhancing DDP against SKOV3 cells. Down-knocking IDO1 augmented the action of DDP in vivo. These data demonstrated that silencing IDO1 enhanced the efficacy of DDP by intensifying p53-dependent apoptosis, and that targeting IDO1 can be a strategy to modulate DDP-based chemotherapy for epithelial ovarian cancer.

## 1. Introduction

Cis-dichlorodiamineplatinum (II) (cisplatin, DDP) is the first-line chemotherapeutic agent in ovarian cancer, which causes DNA damage to induce apoptosis. However, due to the emergence of chemoresistance, 70% of patients with advanced epithelial ovarian cancer suffer from tumor recurrence and treatment failure [1]. Combination chemotherapy is commonly used to re-sensitize the tumor to DDP or to reverse resistance, however, the clinical outcome has been unsatisfactory [2,3]. Therefore, there is an urgent need for chemotherapy modulators. 

Indoleamine 2,3-dioxygenase 1 (IDO1) is an immunosuppressive molecule, which catabolizes tryptophan (Trp) to l-kynurenine (Kyn) [4]. IDO1 induces tumor-related immunosuppression by Trp depletion and Kyn accumulation, which result in dysfunction of natural killer (NK) cells/effector T cells and activate Tregs [4]. Previous data have manifested that IDO1 was highly expressed in multiple cancer tissues including ovarian cancer, and that a high expression level was associated with poor prognosis [5,6,7,8,9]. Okamoto et al. found that 50% of ovarian cancer patients were IDO1-positive and that the overall survival was 11 months in patients with diffuse distribution compared with 41 months survival in patients with sporadic distribution [10]. Niu et al. demonstrated that IDO1 expression was correlated with chemoresistance in high-grade serous carcinoma patients [11]. IDO1 is differentially expressed in DDP-sensitive and -resistant lung cancer cells and affects the sensitivity to DDP [12]. However, the relationship between IDO1 expression and the cellular response to DDP in ovarian cancer, and the underlying molecular mechanisms have been limitedly understood. 

IDO1, a heme-containing dioxygenase, acts by shifting from the resting Fe^3+^-IDO1 form to the active Fe^2+^-IDO1 form, and this process requires reactive oxygen radicals as cofactors to form a heme superoxide adduct [13]. IDO1 exhibits a peroxidase-like function that scavenges reactive oxygen species (ROS) [14]. These data demonstrate the immune-independent function of IDO1 such as regulating ROS level. Notably, DDP induces ROS generation to aggravate DNA damage [15], followed by activation of the p53 signaling pathway and eventual cell death [16]. Recently, Nguyen et al. revealed that inhibiting IDO1 caused ROS accumulation to promote DDP-induced oxidative stress, resulting in cell death [17]. Therefore, IDO1 may impact on the action of DDP through the ROS pathway in ovarian cancer.

The aim of this study was to explore the role of IDO1 in the antitumor efficacy of DDP in epithelial ovarian cancer. IDO1 expression in cancer tissues was detected, and then the function of IDO1 in the cells’ sensitivity to DDP was investigated in vitro and in vivo. Preliminary data indicated that a high expression level of IDO1 was associated with DDP resistance and that inhibiting IDO1 can enhance DDP. The present study represented a novel treatment strategy for sensitizing DDP in epithelial ovarian cancer. 

## 2. Results

### 2.1. A High IDO1 Level in Cancer Tissues Was Associated with Platinum-Resistance and Poor Prognosis in Type II Ovarian Cancer

**Figure 1:**
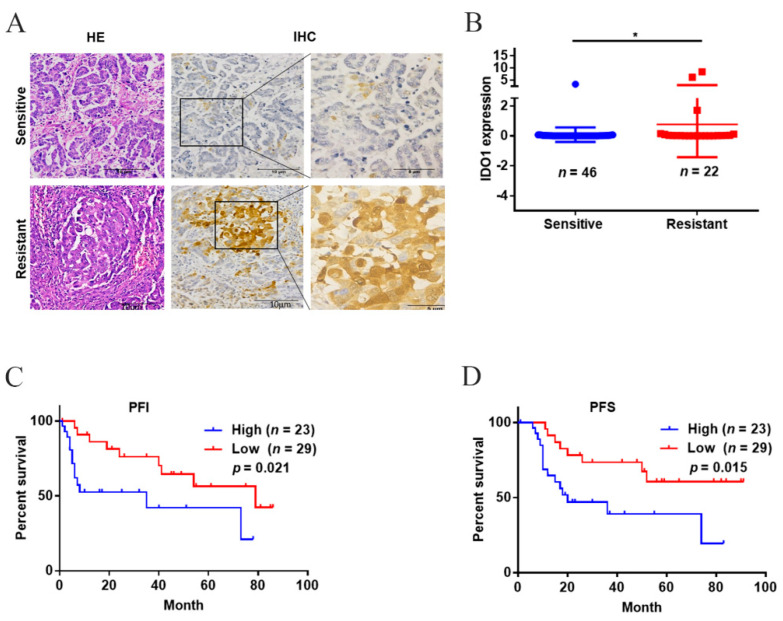
The expression level of IDO1 and the prognosis in patients with ovarian cancer. (**A**) Representative images of IDO1 staining in cancer tissues; the scale bar was 100 μm. (**B**) The IDO1 expression level. (**C**,**D**) Kaplan–Meier plots of PFI and PFS in patients with type II ovarian cancer. Data were mean ± standard deviation. *: *p* < 0.05.

Table 1 illustrated the association between the expression level of IDO1 in cancer tissues and clinicopathological characteristics. The expression level of IDO1 was associated with histological type and platinum-resistance, but was not associated with age, pathological grade, classification, and tumor stage. IDO1 localized in the cytoplasm and the expression level in the platinum-resistant group was higher than that in the -sensitive group (Figure 1A,B). Given that the majority of patients had been high-grade serous carcinoma (i.e., type II ovarian cancer), subtype survival analyses were performed. Patients with a high IDO1 expression level in the tumors had shorter platinum-free interval (PFI) and progression-free survival (PFS) compared with patients having a low level (PFI, median: 34.35 (95% CI: 20.07–48.64) vs. 60.54 (95% CI: 46.19–74.88) months; PFS, median: 39.95 (95% CI: 26.41–53.50) vs. 66.34 (95% CI: 52.53–80.14) months) (Figure 1C,D). These data indicated that a high IDO1 level in the tumors implied lower therapeutic responses and poorer prognosis in type II ovarian cancer. 

### 2.2. A High Level of IDO1 Reduced DDP-Induced Apoptosis via Down-Regulation of the ROS/p53 Pathway

**Figure 2:**
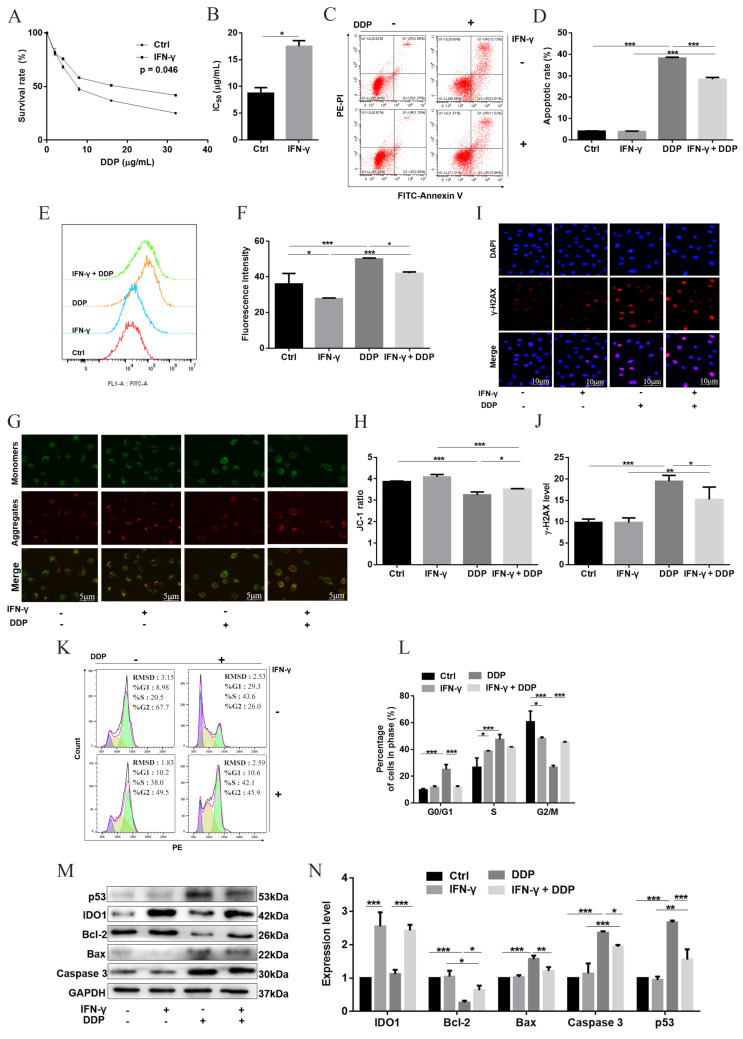
The effect of IDO1 on DDP-induced apoptosis in SKOV3 cells. (**A**,**B**) Cell-survival percentages and IC_50_ of DDP. (**C**,**D**) Apoptotic cells were detected by the Annexin V assay. (**E**,**F**) ROS generation was detected by flow cytometry. Data were expressed as mean fluorescence intensity. (**G**,**H**) MMP determined with the JC-1 assay; representative images of J-aggregates (red) and monomer (green); the scale bar was 5 μm; JC-1 ratio reflected MMP. (**I**,**J**) *γ*-H2AX was detected using immunofluorescence (red), cells were counterstained with DAPI to visualize nuclei (blue); representative images; the scale bar was 10 µm; the fluorescence intensity indicated the *γ*-H2AX level. (**K**,**L**) Cell cycle analyzed by flow cytometry. (**M**,**N**) Apoptosis-related proteins assayed by western blot. Data were mean ± standard deviation for three independent trials. *: *p* < 0.05; **: *p *< 0 .01; ***: *p* < 0 .001.

Both western blot and the Kyn level demonstrated that the expression level of IDO1 was induced by interferon-γ (IFN-γ) in a dose-dependent manner, indicating that IFN-γ treatment can generate IDO1-overexpressing cells (Figure S1B–E). The role of IDO1 in mediating the cellular response to DDP was then investigated. After exposure to serial concentrations of DDP for 24 h, the cell-survival percentage was improved and IC_50_ of DDP was 2.0-fold higher in IDO1-overexpressing cells (group IFN-γ + DDP) than that in IDO1-intact cells (group DDP) (Figure 2A,B). The apoptosis percentage was increased in group DDP, and the percentage in group IFN-γ + DDP was lower than that in group DDP (Figure 2C,D). The ROS level was slightly decreased in IDO1-overexpressing cells (group IFN-γ) compared with control cells, and the level of DDP-induced ROS was reduced in cells overexpressing IDO1 (Figure 2E,F). *γ*-H2AX level was determined, which was a molecular marker of DNA double-strand break (DSB) [18]. A similar trend as apoptosis was observed for *γ*-H2AX and MMP (Figure 2G–J). Subsequently, cell cycle distribution was assessed. The percentages of G0/G1- and S-phase cells in group DDP were higher than those in group Ctrl; the percentage of G0/G1-phase cells was decreased and that of G2/M-phase cells was increased in group IFN-γ + DDP, compared with group DDP (Figure 2K,L). Thus, over-expressing IDO1 led to G2/M arrest and promoted DNA repair. Finally, apoptosis-related proteins were analyzed. Levels of p53, Bax, and caspase 3 in group IFN-γ + DDP were lower than those in group DDP, but with a higher level of Bcl-2 (Figure 2M,N). These data demonstrated that a high level of IDO1 decreased cytotoxicity of DDP; this was realized through down-regulation of the ROS/p53 pathway, thereby reducing DDP-induced apoptosis. 

### 2.3. Silencing IDO1 Enhanced DDP through Activation of ROS/p53-Dependent Apoptois in SKOV3 Cells

**Figure 3:**
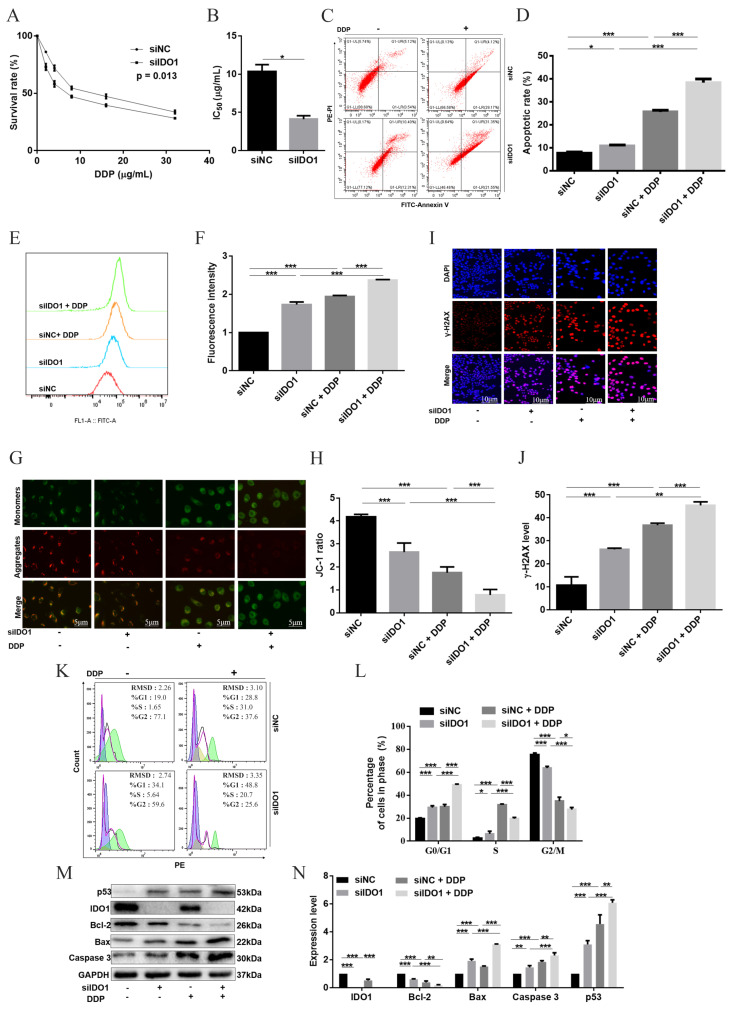
Silencing IDO1 enhanced DDP-induced apoptosis in SKOV3 cells. (**A**,**B**) Cell-survival percentages and IC_50_ of DDP. (**C**,**D**) Apoptosis was determined by the Annexin V assay. (**E**,**F**) ROS generation was detected by flow cytometry. Data were expressed as mean fluorescence intensity. (**G**,**H**) MMP determined with the JC-1 assay; representative images of J-aggregates (red) and monomer (green); the scale bar was 5 μm; JC-1 ratio reflected MMP. (**I**,**J**) DNA damage marker *γ*-H2AX assayed with immunofluorescence (red), cells were counterstained with DAPI to visualize nuclei (blue); representative images; the scale bar was 10 µm; the fluorescent intensity indicated the *γ*-H2AX level. (**K**,**L**) Cell cycle examined by flow cytometry. (**M**,**N**) Apoptosis-related proteins analyzed by western blot. Data were mean ± standard deviation for 3 independent trials. *: *p* < 0.05; **: *p* < 0 .01; ***: *p* < 0 .001.

To further verify the involvement of IDO1 in the cells’ response to DDP, IDO1 was knocked down using siRNA (Figure S2A,B). Then, cells were treated with DDP with a series of concentrations for 24 h; IC_50_ of DDP was decreased in IDO1-knockdown cells compared with cells transfected with siNC (Figure 3A,B). The apoptosis percentage was elevated in groups siIDO1 + DDP and siNC + DDP, with a higher level in group siIDO1 + DDP (Figure 3C,D). Down-knocking IDO1 enhanced ROS accumulation, MMP collapse and DNA damage due to DDP (Figure 3E–J). More G0/G1 cells, and less S and G2/M cells were observed in group siIDO1 + DDP, compared with group siNC + DDP (Figure 3K,L). For apoptotic proteins, levels of p53, caspase 3, and Bax were increased in group siIDO1 + DDP in comparison with group siNC + DDP, but the Bcl-2 level was decreased (Figure 3M,N). These data indicated that silencing IDO1 enhanced DDP against ovarian cancer cells via up-regulation of ROS/p53-dependent apoptosis. 

### 2.4. Inhibition of IDO1 Improved the Anticancer Efficacy of DDP In Vivo

**Figure 4:**
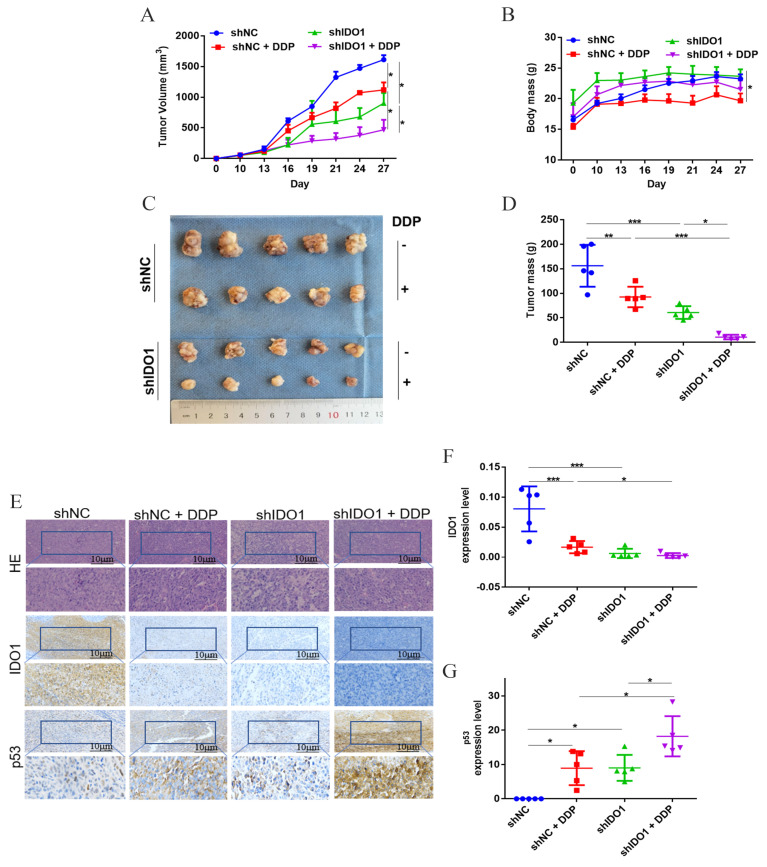
Silencing IDO1 enhanced action of DDP in vivo. (**A**) The tumor volume vs. day curve. (**B**) Alteration in the body mass of mice. (**C**,**D**) Macroscopic tumors and tumor mass after treatments. (**E**–**G**) Expression of IDO1 and p53 in tumor tissues; the scale bar was 10 µm. Data were mean ± standard deviation for 5 mice. *: *p* < 0.05; **: *p* < 0 .01; ***: *p* < 0 .001.

To better understand the impact of IDO1 on efficacy of DDP, in vivo therapies were conducted. shNC/shIDO1-transfected cells were injected into nude mice to form tumors. Tumor growth was inhibited from day 16 in groups shIDO1 and DDP, and the most severe suppression was noted in group shIDO1 + DDP (Figure 4A). Similarly, the tumor mass in group shIDO1 + DDP was lower than that in group shIDO1 or DDP (Figure 4C,D). During treatments, the body mass of mice did not decrease in group shIDO1, suggesting that knockdown of IDO1 produced no obvious toxicity (Figure 4B). IHC was performed to analyze the expression of IDO1 and p53 in tumor tissues. IDO1 localized in the cytoplasm being consistent with observations in clinical cancer tissues; p53 was expressed in both the cytoplasm and nucleus. The expression level of IDO1 was down-regulated upon IDO1 knockdown and/or DDP treatment, but the level of p53 was up-regulated (Figure 4E–G). These data indicated that silencing IDO1 inhibited tumor growth and enhanced the anticancer effect of DDP in vivo.

## 3. Discussion

The present study explored the relationship between IDO1 and the DDP efficacy in ovarian cancer. A high expression level of IDO1 was associated with poorer platinum responses and shorter PFS/PFI in patients with type II cancer. IDO1 regulated apoptosis through the ROS/p53 pathway, thereby modulating the cells’ response to DDP.

Preclinical data demonstrated that IDO1 strengthened the immune escape of tumors and that targeting IDO1 suppressed tumor growth in ovarian cancer [19,20]. However, the antitumor effects of an IDO1 inhibitor alone were not confirmed [21]. Thus, the non-immune role of IDO1 in the cancer microenvironment should be considered. Here, approximately 51.5% of ovarian cancer patients had high expression of IDO1 in cancer tissues, and a low expression level indicated longer PFS and PFI in type II cancer, which was in agreement with the findings of Taka et el. [22]. Additionally, 72.7% of platinum-resistant patients had a high IDO1 level, and alterations in IDO1 expression impacted the response to DDP in SKOV3 cells. Therefore, IDO1 was a determinant for the therapeutic outcome in ovarian cancer patients receiving platinum-based chemotherapy. 

IFN-γ-induced IDO1 up-regulation appeared after 2–5 h, and can maintain –5 days after removing IFN-γ [23]. Here, IFN-γ was washed away after 24 h, and then cells were exposed to DDP; this sequential mode avoided interferences of IFN-γ, i.e., the cellular responses were due to IDO1 and/or DDP. IDO1 decreased DDP-induced apoptosis by reducing the intracellular ROS level, thereby alleviating the cytotoxicity of DDP. Conversely, silencing IDO1 increased ROS accumulation, intensified apoptosis, and ultimately enhanced the action of DDP. IDO1 was critical for regulating intracellular ROS [24,25,26]. ROS overproduction caused DNA damage and exacerbated DNA damage due to DDP, which triggered the p53 signaling pathway in apoptosis and cell cycle arrest [27,28]. Active p53 up-regulated the transcription of Bax, increased the ratio of Bax/Bcl-2 leading to MMP collapse, and eventually activated caspase 3 to realize apoptosis [29,30]. γ-H2AX demonstrated the formation of DSB after DDP exposure, and the damage degree was reliant on the IDO1 level; a higher level reduced DSB but a low level aggravated DSB. Unrepairable DSB triggered apoptosis. Thus, IDO1 acted on DNA damage, thereby modulating the cells’ sensitivity to DDP.

Whether IDO1-related DNA damage altered the expression of p53 and p53 downstream genes had not yet been investigated. Silencing IDO1 inhibited tumor cell growth by upregulating p53, coupled with increases in p53 upregulated modulator of apoptosis (PUMA) and Bax in B-cell lymphoma [31]. Liu et al. revealed that IDO1 was related to the Bcl-2/Bax pathway in IDO1-overexpressing HeLa cells [32]. In this study, overexpressing IDO1 down-regulated the DDP-induced p53 signal pathway, thereby decreasing Bax and caspase 3 expression and increasing Bcl-2 expression; in contrast, silencing IDO1 up-regulated the p53-dependent apoptosis signal. IDO1 affected p53-related cell cycle arrest. Overexpressing IDO1 enhanced G2/M phase cell-cycle arrest, thereby improving DNA repair to promote DDP resistance. Down-knocking IDO1 enhanced G0/G1 phase cell-cycle arrest, inhibited cell cycle progression and ultimately caused cell death, thereby enhancing the efficacy of DDP. IDO1 was associated with G0/G1 phase arrest [31], and the IDO1 inhibitor 1-L-MT caused G2/M cell cycle block and prevented mitosis in rectal cancer cells [33]. DDP treatment resulted in S, G2/M cell-cycle arrest in ovarian cancer cells [34,35], and here silencing IDO1 arrested more cells in G0/G1 and S phases. Consequently, the combination of IDO1-silence and DDP inhibited cells in each phase, leading to synergism. 

In vivo data demonstrated that silencing IDO1 suppressed tumor growth and potentiated the anticancer action of DDP. The expression level of IDO1 in tumors was decreased in groups shIDO1, shNC + DDP, and shIDO1 + DDP, but with a higher p53 level. These confirmed that IDO1 modulated the cells’ responses to DDP via the p53 pathway, and therefore it was worthwhile to test IDO1 inhibitors (small molecules or antibodies) in ovarian cancer. IDO1 expression was down-regulated in tumors in group shNC + DDP, which was not observed in vitro. The discrepancy may be due to the cancer microenvironment and play a part in the low therapeutic efficacies of IDO1 inhibitors noted in previous clinical trials. Further investigations were needed to elucidate the function of IDO1. 

In summary, a high expression level of IDO1 in tumors was associated with DDP resistance and poor prognosis in type II ovarian cancer patients. IDO1 decreased the cellular response to DDP, and knockdown of IDO1 enhanced the action of DDP in vitro and in vivo via ROS/p53 apoptosis pathway. Thus, IDO1 can be a target for modulating the efficacy of DDP-based chemotherapy.

## 4. Materials and Methods

### 4.1. Patients and Cancer Samples

Paraffin-embedded ovarian cancer samples from 68 patients who underwent primary cytoreductive surgery followed by platinum-based chemotherapy from January 2013 to March 2020 were obtained from the Second Affiliated Hospital, Chongqing Medical University (Chongqing, China). Clinical characteristics were noted. The response to platinum therapy was determined according to the recurrence or progression interval from the last dose; an interval of ≥6 months was considered sensitive, and an interval of <6 months was resistant [36]. There were 42 sensitive cases and 26 resistant cases. PFS (i.e., the interval from surgery to disease progression) and PFI (i.e., the interval between the last platinum-based chemotherapy and disease progression) were used to assess the clinical outcome. The use of human tissues was ethically approved by the Institutional Review Board.

### 4.2. Detection of IDO1 in Cancer Tissues with an Immunohistochemistry (IHC) Assay

IDO1 in cancer tissues was immunohistochemically detected using a streptavidin-peroxidase kit (ZSGB-BIO, Beijing, China). Images were analyzed with the software Image-Pro Plus (Media Cybernetics, Rockville, MD, USA), and the expression level of IDO1 was quantified with the mean density.

### 4.3. Cells

Human epithelial ovarian cell line SKOV3 (identified by STR; Cell Bank, Type Culture Collect., Chin. Acad. Sci., Shanghai, China) was cultured in RPMI 1640 medium (Gibco, Beijing, China), supplemented with 10% fetal bovine serum (Biol. Ind., Kibbutz, Beit Haemek, Israel) and 1% penicillin-streptomycin (Beyotime Biotechnol, Shanghai, China), at 5% CO_2_ and 37 °C.

### 4.4. Cell Transfection

For transient transfection, 100 pmol of IDO1 small interfering RNA (siRNA) (GenePharma, Shanghai, China) or negative control (NC) was transfected into SKOV3 cells with GP-transfect-Mate reagent (GenePharma). Lentiviral shRNA (Genechem, Shanghai, China) was employed for stable transfection; 2 μg/mL puromycin (Beyotime) was added to the medium to remove uninfected cells. The knockdown efficiency was validated by western blot. siRNA-transfected cells were used for in vitro experiments and shRNA-infected cells were adopted for establishing subcutaneous tumors in vivo. The siRNA and shRNA sequences were listed in Tables S1 and S2.

### 4.5. In Vitro Treatments

IFN-γ was used to up-regulate the expression level of IDO1 [23]. Cells were seeded in a 96-well plate at a density of 5.0 × 10^3^ cells per well, and were exposed to IFN-γ (0, 2.0, 4.0, 8.0, 16.0, and 32.0 ng/mL) (Cell Signal. Technol., Danvers, MA, USA) or DDP (0, 2.0, 4.0, 8.0, 16.0, and 32.0 µg/mL) (Yunnan Phytopharm., Kunming, China) for 24 h. This was to determine the drug concentration for in vitro experiments. 

For in vitro therapies, cells were exposed to 10 ng/mL IFN-γ for 24 h (group IFN-γ); 10 ng/mL IFN-γ for 24 h followed by 6 µg/mL DDP for another 24 h (group IFN-γ + DDP); or 6 µg/mL DDP for 24 h (group DDP). Control cells received iso-volumetric medium.

SiRNA-infected cells were divided into 4 groups: siNC group; siNC + DDP group; siIDO1 group; and siIDO1 + DDP group. Groups siNC + DDP and siIDO1 + DDP were administrated 6 µg/mL DDP, and other groups received iso-volumetric medium. 

### 4.6. Cell Viability

A CCK-8 assay (Dojindo, Kumamoto, Japan) was used to determine cell viability, and the percentage of surviving cells was calculated.

### 4.7. Apoptosis and Cell Cycle Analyses

Apoptotic cells were detected using the Annexin V assay (Nanjing Keygen Biotech., Nanjing, China), and cell cycle was analyzed with a kit (Beyotime). 

### 4.8. Intracellular ROS

The intracellular ROS level was measured using the dichloro-dihydro-fluorescein diacetate (DCFH-DA) assay (Beyotime).

### 4.9. Mitochondrial Membrane Potential (MMP)

MMP was measured using the JC-1 assay (Beyotime). Cells were observed under a fluorescence microscope (Nikon, Tokyo, Japan). The fluorescence intensity was analyzed by the software ImageJ (NIH, Bethesda, MD, USA), and the ratio of red to green fluorescence reflected the MMP [37].

### 4.10. Determination of Kyn in Culture Supernatants

The culture supernatants were collected and mixed in a 1:3 ratio with 30% trichloroacetic acid (Macklin, Shanghai, China). The mixture was incubated at 60 °C for 50 min, and centrifuged (11,500 rpm, 4 °C, 15 min). 2% p-dimethylaminobenzaldehyde (Macklin) dissolved in glacial acetic acid was added to the supernatant (1:1). Absorbance was measured at 480 nm. The concentration of Kyn was determined and normalized to the total protein concentration, which indicated the IDO1 enzymatic activity [38].

### 4.11. Determination of γ-H2AX by Immunofluorescence

Cells were fixed with 4% paraformaldehyde for 30 min, permeabilized with 0.1% Triton X-100 for 30 min, and blocked with 10% bovine serum albumin for 1 h at room temperature. Then, cells were incubated with an anti-*γ*-H2A.X antibody (ABclonal, Wuhan, China) at 4 °C overnight, followed by incubation with goat anti-rabbit IgG antibody (Alexa Fluor 594-conjugated; ABclonal) for 1 h at room temperature. Nuclei were counterstained with 4′,6-diamidino-2-phenylindole (DAPI; Beyotime). Cells were observed under a fluorescence microscope (Nikon), and the fluorescent intensity was measured using the software ImageJ 1.53k.

### 4.12. Western Blot

Western blot was performed to detect proteins, including IDO1, Bcl-2, Bax (Bcl-2 associated X protein), caspase 3, and p53. The primary antibodies were listed in Table S3, and the secondary antibody was a goat-anti-rabbit IgG antibody conjugated to horseradish peroxidase (diluted 1:15,000; Abcam, Cambridge, UK). GAPDH was used as the loading control. Proteins were visualized using an enhanced chemiluminescence kit (Millipore, Burlington, MA, USA). Bands were analyzed using the software Image Lab 6.0 (Bio-Rad Lab., Hercules, CA, USA).

### 4.13. In Vivo Therapies on Subcutaneous Tumors in Nude Mice

All animal procedures were ethically approved by the Local Review Board in compliance with the Guide for the Care and Use of Laboratory Animals.

Female BALB/c nude mice (4–6 weeks old, 15–20 g, Ctr. Lab. Anim., Chongqing Med. Univ.) were used for the in vivo studies. shNC/shIDO1-transfected SKOV3 cells (1.0 × 10^7^) were injected into the scapula region and the tumors were allowed to reach a 50 mm^3^ volume after about 10 days, following which the animals were randomly divided into the 4 groups: shNC; shIDO1; shNC + DDP; and shIDO1 + DDP. DDP (10 mg/kg) was injected through the tail vein every 4 days, and 4 cycles were administrated [39]. Body mass and the tumor volume (length × width^2^/2) were measured every 3 days. Mice were euthanized at the end of the experiment, tumor volume and mass were determined, and the expression levels of IDO1 and p53 in tumor tissues were analyzed with an IHC assay.

### 4.14. Statistical Analysis

Statistical analyses were performed using the software SPSS 26.0 (SPSS Inc., Chicago, IL, USA) and GraphPad Prism 6 (GraphPad Software, San Diego, CA, USA). The chi-square test was used to assess the association of IDO1 expression level with clinical variables. The cutoff value for IDO1 expression level (high/low) in cancer tissues was determined using the receiver operating characteristic curve. Differences were analyzed by the Student’s t test or analysis of variance. PFI and PFS were analyzed by the Kaplan–Meier method. *p* < 0.05 was defined as statistically significant. 

## Figures and Tables

**Table 1:** Correlation between clinicopathological parameters and the level of IDO1 in ovarian cancerous tissues.

Clinicopathological Features	Case No.	IDO1 Expression Level		*p* Value
		Low (*n* = 33)	High (*n* = 35)	
Age (year)				0.492
<50	24	13 (54.2%)	11 (45.8%)	
≥50	44	20 (45.5%)	24 (54.5%)	
Histological type				0.012 *
Serous	54	22 (40.7%)	32 (59.3%)	
Others	14	11 (78.6%)	3 (21.4%)	
Pathological grade				0.447
G1	2	2 (100.0%)	0 (0.0%)	
G2 + G3	66	31 (47.0%)	35 (53.0%)	
Classification				0.201
Type I	16	10 (62.5%)	6 (37.5%)	
Type II	52	23 (44.2%)	29 (55.8%)	
FIGO stage				0.140
I/II	21	13 (61.9%)	8 (38.1%)	
III/IV	47	20 (42.6%)	27 (57.4%)	
Platinum response				0.015 *
Resistant	22	6 (27.3%)	16 (72.7%)	
Sensitive	46	27 (58.7%)	19 (41.3%)	

FIGO: International Federation of Gynecology and Obstetrics; *: *p* < 0.05.

## Data Availability

The data presented in this study are available on request from the corresponding author. The data are not publicly available due to privacy or ethical restrictions.

## Supplementary Materials

The following supporting information can be downloaded at: https://www.mdpi.com/article/10.3390/ijms231912002/s1.

## Abbreviations

DAPI	4′,6-diamidino-2-phenylindole
DDP	cis-dichlorodiamineplatinum (II), cisplatin
DSB	double-strand *DNA* break
Bax	Bcl-2 associated X protein
IDO1	indoleamine 2,3-dioxygenase 1
IFN-γ	interferon-γ
IHC	immunohistochemistry
Kyn	l-kynurenine
PFI	platinum-free interval
PFS	progression-free survival
PUMA	p53 upregulated modulator of apoptosis
ROS	reactive oxygen species
Trp	tryptophan
