# Supplementary material for: IDO1 Modulates the Sensitivity of Epithelial Ovarian Cancer Cells to Cisplatin through ROS/p53-Dependent Apoptosis
Source: Int J Mol Sci. 2022 Oct 9;23(19):12002. doi: 10.3390/ijms231912002 (PMC9569641; doi:10.3390/ijms231912002)
Supplement: Supplementary file 1 [file ijms-23-12002-s001.zip › supplementary Fig .pdf]

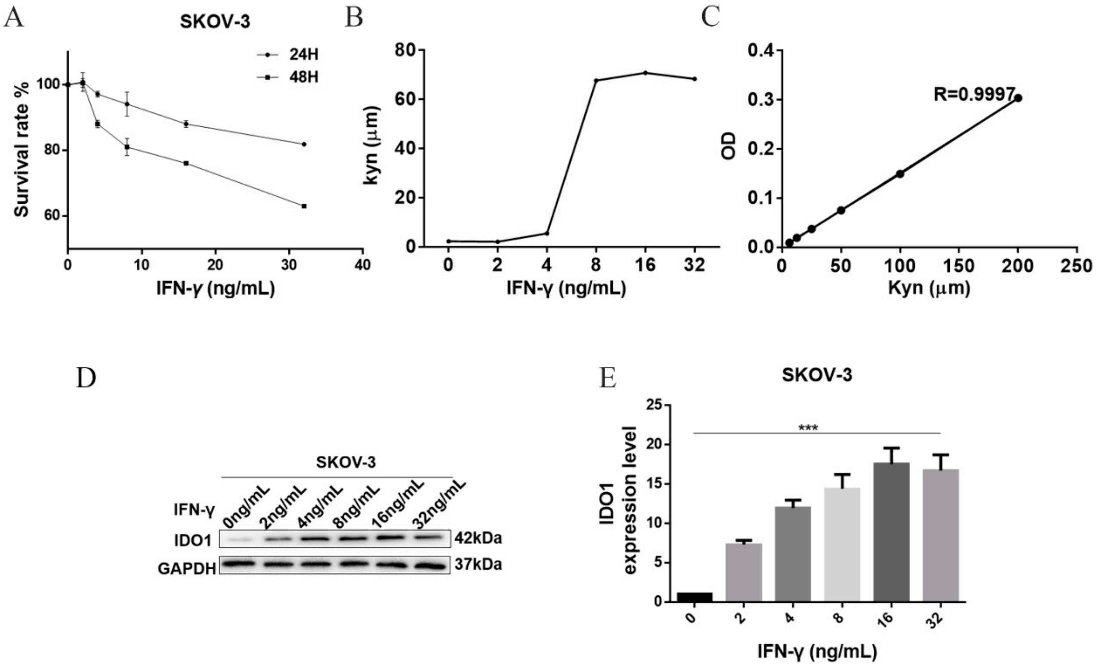

Supplementary Figure S1 The effect of IFN-γ on IDO1 induction and the concentrations of Kyn in SKOV3 cells. The cell viability was significantly inhibited with IFN-γ treatment for 48h in a dose-dependent manner, and the survival rate of the cell was less than 80% when the concentration was more than 8 ng/mL. To reduce the interference of IFN-γ, the cells were proposed for pretreatment with IFN-γ for 24 h. Incubated with IFN-γ for 24 h, the cell survival rate was less than 90% at the dose of IFN-γ above 16 ng/mL. In addition, IDO1 expression was induced by IFN-γ in a dose-dependent manner when the dose of IFN-γ was more than 2 ng/mL. Accordingly, the Kyn content in the cell supernatant increased at the dose of IFN-γ above 4 ng/mL and peaked at the dose of 8 ng/mL, which indicated that the enzyme activity of IDO1 was maximal. Taking into account the expression and activity of IDO1,

10 ng/mL IFN-γ was used for pre-treatment. Finally, pre-treatment with 10 ng/mL IFN-γ for 24 h was applied as the experimental condition. (A) Survival rate after exposure to various concentrations of IFN-γ (0, 2.0, 4.0, 8.0,16.0 and 32.0 ng/mL) for 24 or 48 hours in SKOV3 cells. (B) The concentrations of Kyn after exposure to various concentrations of IFN-γ (0, 2.0, 4.0, 8.0,16.0 and 32.0 ng/mL) for 24 hours. (C) A standard curve was drawn according to the OD480 value and the concentrations of Kyn standards (30, 60, 120, 240 and 480 μM, R = 0.9997). (D–E) Western blot for IDO1 expression after exposure to various concentrations of IFN-γ (0, 2.0, 4.0, 8.0,16.0 and 32.0 ng/mL) for 24 hours in SKOV3 cells. ***: $p < 0.01$.

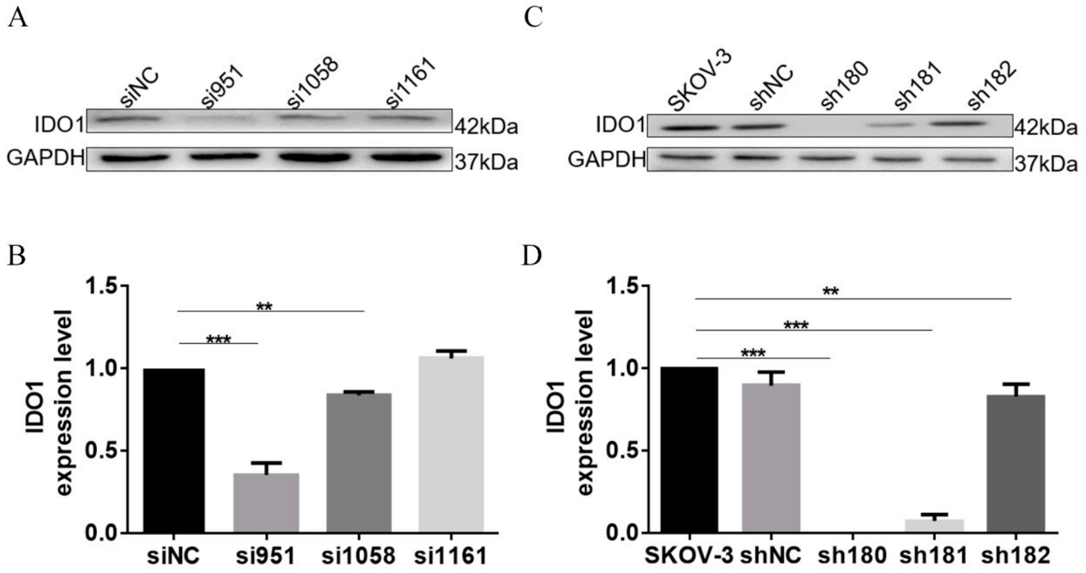

Supplementary Figure S2 Efficiency of silencing IDO1 expressing in SKOV3 cells (A,B) For transient transfection,100 pmol small interfering RNA (siRNA) for IDO1 or negative control was transfected into SKOV3 cells with GP-

transfect-Mate transfection reagent. After 72 h, silencing efficiency was assessed by Western blot with GAPDH as a loading control. The expression of IDO1 was knocked down by approximately 70% in siRNA#951 transfected cells compared to negative control cells ($p < 0.001$), and then siRNA#951 was used for further experiments. (C,D) IDO1 and negative lentiviral shRNA were transfected into SKOV3 cells (MOI = 10 for 72h). The knockdown efficiency was also validated by Western blot. IDO1 expression was almost extinguished in shRNA#180 transfected cells, which were used for further experiments ($p <$ 0 .001). **: $p < 0.01$; ***: $p < 0.001$.
