# Supplementary material for: IDO1 Modulates the Sensitivity of Epithelial Ovarian Cancer Cells to Cisplatin through ROS/p53-Dependent Apoptosis
Source: Int J Mol Sci. 2022 Oct 9;23(19):12002. doi: 10.3390/ijms231912002 (PMC9569641; doi:10.3390/ijms231912002)
Supplement: Supplementary file 1 [file ijms-23-12002-s001.zip › supplementary table.pdf]

Supplementary Table S1 RNA oligo sequence of siRNA

NO.	Target Seq	Source
Negative control	Sense 5′_UUC UCC GAA CGU GUC ACG UTT_3′<br>Antisense 5′_ACG UGA CAC GUU CGG AGA ATT _3′	GenePharma
IDO1-Homo-951	Sense 5′_GUG CUC AUU AGA GUC AAA UTT_3′<br>Antisense 5′_AUU UGA CUC UAA UGA GCA CTT _3′	GenePharma
IDO1-Homo-1058	Sense 5′_GGA GCU ACC AUC UGC AAA UTT_3′<br>Antisense 5′_AUU UGC AGA UGG UAG CUC CTT _3′	GenePharma
IDO1-Homo-1161	Sense 5′_GAG GCA CUG AUU UAA UGA ATT_3′<br>Antisense 5′_UUCAUU AAA UCA GUG CCU CTT_3′	GenePharma

Supplementary Table S2 RNA oligo sequence of shRNA

NO.	Target Seq	Source
CON313	TTCTCCGAACGTGTCACGT	Genechem
IDO1-RNAi(107180-1)	ccATCTGCAAATCGTGACTAA	Genechem
IDO1-RNAi(107181-1)	gcCCTTCAAGTGTTTCACCAA	Genechem
IDO1-RNAi(107182-2)	cgTAAGGTCTTGCCAAGAAAT	Genechem

Supplementary Table S3 Primary Antibodies

Name	Dilution ratio	Assay	Source	NO.
IDO1 Rabbit mAb	1:800	WB	CST	#51851
	1:300	IHC		
p53 Rabbit mAb	1:800	WB	CST	#2527
	1:250	IHC		
Caspase 3 Rabbit mAb	1:100	WB	CST	#9662
Bax Rabbit mAb	1:100	WB	ABclonal	#A18642
Bcl-2 Rabbit mAb	1:100	WB	ABclonal	#A19693
Phospho-Histone γ-H2AX-S139 Rabbit mAb	1:200	IF	ABclonal	#Ap0687
GAPDH Rabbit mAb	1:4000	WB	Bioss	#0755R
